# Leptin in Whales: Validation and Measurement of mRNA Expression by Absolute Quantitative Real-Time PCR

**DOI:** 10.1371/journal.pone.0054277

**Published:** 2013-01-16

**Authors:** Hope C. Ball, Robert K. Holmes, Richard L. Londraville, Johannes G. M. Thewissen, Robert Joel Duff

**Affiliations:** 1 Department of Biology, The University of Akron, Akron, Ohio, United States of America; 2 Department of Anatomy and Neurobiology, Northeast Ohio Medical University, Rootstown, Ohio, United States of America; Università degli Studi di Milano, Italy

## Abstract

Leptin is the primary hormone in mammals that regulates adipose stores. Arctic adapted cetaceans maintain enormous adipose depots, suggesting possible modifications of leptin or receptor function. Determining expression of these genes is the first step to understanding the extreme physiology of these animals, and the uniqueness of these animals presents special challenges in estimating and comparing expression levels of mRNA transcripts. Here, we compare expression of two model genes, leptin and leptin-receptor gene-related product (OB-RGRP), using two quantitative real-time PCR (qPCR) methods: “relative” and “absolute”. To assess the expression of leptin and OB-RGRP in cetacean tissues, we first examined how relative expression of those genes might differ when normalized to four common endogenous control genes. We performed relative expression qPCR assays measuring the amplification of these two model target genes relative to amplification of 18S ribosomal RNA (18S), ubiquitously expressed transcript (Uxt), ribosomal protein 9 (Rs9) and ribosomal protein 15 (Rs15) endogenous controls. Results demonstrated significant differences in the expression of both genes when different control genes were employed; emphasizing a limitation of relative qPCR assays, especially in studies where differences in physiology and/or a lack of knowledge regarding levels and patterns of expression of common control genes may possibly affect data interpretation. To validate the absolute quantitative qPCR methods, we evaluated the effects of plasmid structure, the purity of the plasmid standard preparation and the influence of type of qPCR “background” material on qPCR amplification efficiencies and copy number determination of both model genes, in multiple tissues from one male bowhead whale. Results indicate that linear plasmids are more reliable than circular plasmid standards, no significant differences in copy number estimation based upon background material used, and that the use of ethanol precipitated, linearized plasmid preparation produce the most reliable results.

## Introduction

Leptin is a small, 16 kDa peptide hormone encoded by the obese (*ob*) gene in vertebrates [1]. Leptin was originally characterized as a “lipostat” involved in regulation of body fat stores; synthesized primarily by adipose cells and secreted into the blood where it circulates to bind to receptors (obese gene receptors or OBRs) found in the hypothalamus [2]. Once bound, leptin stimulates transcription of genes up-regulating metabolic rate and lipid catabolism and down regulating appetite [2]. In this fashion, the brain monitors and balances lipid stores through the regulation of appetite and metabolism. Leptin-receptor gene related product (OB-RGRP) is a product linked via alternative splicing to the leptin receptor [3]. Recent work in mice suggests that OB-RGRP regulates post-translational Golgi processing of the leptin receptor itself, regulating the number of receptor copies functional at the cell surface making it a possible mechanism of temporal and tissue-specific regulation of receptor function [4], [5].

The majority of leptin studies have focused on the role of the protein in lipid metabolism and energy homeostasis in model mammals (mice and rats) and humans. Leptin is pleiotrophic in its effects and its effect on obesity are well documented in rodent models. Rodents deficient in either leptin [1] or the leptin receptor [6] are obese and restoration of the leptin signal normalizes weight. Additionally, examinations of several hibernating and migrating mammals have demonstrated increased leptin titer prior to their seasonal migration or hibernation [7], [8], [9], [10], [11]. It has been hypothesized that temporary leptin resistance, possibly through sequestering of leptin via circulating leptin-binding proteins, may play a role in reducing the anorexigenic effects of leptin [11]. Obese humans are considered *leptin resistant*, meaning they are capable of making abundant leptin but downstream receptors do not respond to this large leptin signal as it does in people with normal body weight [12]. The two marine mammals examined in this study, build and maintain large subcutaneous fat reserves (blubber) and questions remain as to whether or not this requires leptin resistance. Subcutaneous adipose depots in whales function in locomotion, energy storage, maintenance of water balance and insulation, where increases in blubber thickness allows for a wider temperature range to be obtained without alterations to the animal’s metabolic rate [13]. This insulating capacity of blubber is vital for arctic adapted cetaceans such as bowhead (*Balaena mysticetus*) and beluga (*Delphinapterus leucas*) whales that continually inhabit ≤1.8°C Arctic seas. Both species produce a thick epidermis in addition to blubber and adult bowheads in particular have blubber that may reach up to 50 cm in depth making those depots the largest known in any mammal [14]. Knowing that blubber is the principal source of leptin production in whales is useful, but is their leptin expression unusual compared to other mammals with more modest adipose stores?

Quantitative real-time PCR (qPCR) methodology was developed to allow for highly sensitive and reproducible measurements of relative abundance of mRNA transcripts from a wide variety of species and samples; providing an alternative to more time-consuming traditional methods of gene expression such as RNase protection assays, Northern blots or competitive quantitative PCRs [15], [16], [17]. Two forms of qPCR, “relative” and “absolute” have emerged as viable methods for the quantification of a target gene. ‘Relative’ qPCR is the most commonly used method whereby amplification of a target gene is normalized to reduce variation between samples through the utilization of an exogenous internal standard added to the samples or, more commonly, an endogenous internal standard. A potential weakness in the use of endogenous controls is the assumption that the control gene selected demonstrates no significant variation in expression from one tissue to the next. In fact, several studies have reported significant variations in the expression of several commonly used endogenous controls of several species raising questions about how this might impact relative expression data analyses [18], [15], [19], [20], [21]. Relative qPCR is also limited because comparisons made among samples run on multiple qPCR plates must include suitable standards in each assay, and results do not yield actual copy numbers for target genes. More importantly, when relative abundance assays are conducted on non-model organisms, where validation of endogenous control genes is problematic, differences in physiology and/or a lack of knowledge regarding the expression levels and patterns of control genes may impact results.

A second, less common, method for analysis of target gene expression is ‘absolute’ quantitative real-time PCR, which shares the highly sensitive and reproducible methods of detection commonly found in relative qPCR assays but differs by not employing the use of an endogenous internal control gene. Absolute qPCR assays allow for quantification of target template copy numbers based on the construction of a standard curve from PCR amplified products, cDNA/DNA, oligonucleotides or plasmid DNA sources [22]. While requiring more up front preparation, this powerful technique permits comparisons from the same organism and across species. Like relative qPCR, though, the reliability and validity of the method is based on a number of assumptions. For example, the most commonly used method for creating a standard curve is the use plasmid DNA, where the circular structure of the plasmid is thought to provide increased stability. However, questions have recently arisen about what affect this circular (mostly supercoiled) structure has on qPCR amplification efficiencies [22], [6], [23]. Hou, *et al*., recently demonstrated significant overestimations in copy number for the gene *pcna* in several species of microalgae when whole circular plasmid standards were used to compare copy numbers estimations from linearized versions of the same plasmid standards [22]. This known problem, in addition to others is further examined in this paper using two genes (leptin and leptin-receptor gene related product (OB-RGRP) as model genes to demonstrate the technique.

To assess the expression of leptin and OB-RGRP in cetacean tissues, we first examined how relative expression of those genes might differ when normalized to four common endogenous control genes. We performed relative expression qPCR assays measuring the amplification of two target genes (leptin and OB-RGRP) relative to amplification of 18S ribosomal RNA, ubiquitously expressed transcript (Uxt), ribosomal protein 9 (Rs9) and ribosomal protein 15 (Rs15) endogenous controls. Relative expressions of both target genes were then analyzed following the 2^ΔΔCT^ method described by Bustin [15]. The results demonstrate that none of the four common endogenous controls were stably expressed in cetacean tissues, emphasizing the weaknesses of relative qPCR due to assumptions in the methods. To further compare endogenous control expression, we obtained absolute quantification data for leptin and OB-RGRP expression data from several tissues of four bowhead and three beluga whales as well as copy number determinations of four common endogenous control genes (18S, Uxt, Rs9 and Rs15) and discuss how these data can inform us about individual and possible sex-specific differences in these genes.

To validate the absolute quantitative qPCR methods used to quantify cetacean leptin and OB-RGRP gene expression we evaluated the effects of plasmid structure (circular and linearized plasmid), the purity of the plasmid standard preparation (precipitated versus un-precipitated linearized standards) and the influence of type of qPCR “background” material (RNA or cDNA) on qPCR amplification efficiencies and copy number determination of both model genes in various tissues from one male bowhead whale (09B9). qPCR amplification efficiencies and any significant differences observed in threshold cycle (Ct) values and copy numbers were determined and compared for each test and each gene. Findings from this study strongly support previous findings that linear plasmids are more reliable than circular plasmid standards. In addition we demonstrate that no significant differences were seen in copy number estimation based upon background material used (RNA or cDNA) and that use of ethanol precipitated, linearized plasmid preparations in qPCR assays provides the most reliable results.

## Materials and Methods

### Sample Acquisition

Tissue samples were acquired for bowhead (*Balaena mysticetus*) and beluga (*Delphinapterus leucas*) whales by J.G.M. Thewissen (permit: NOAA-NMFS 814–1899). The collection permit follows the provisions of the Marine Mammal Protection Act of 1972 as amended (MMPA: 16 U.S.C 1361 et seq) as well as the Endangered Species Act of 1973 as emended (ESA 16 U.S.C 1531 et seq). Post-mortem samples of, liver, whole heart muscle, and testes were taken from one male bowhead (2009B9) approximately 5–10 years of age. Post-mortem samples of liver, whole heart tissue and reproductive tissue were taken from a three female bowheads (2009B7, 2010B15 and 2011B3) approximately 7–12 years for the former and greater than 20 years of age for the later two, an additional male bowhead (10B16) approximately 2 years of age and beluga whales 2009LDL26 (9 years of age), 2010LDL17 (15 years of age) and 2010LDL5 (13 years of age). Age estimations for bowhead whales were done through examination of baleen length [24]. Bowhead whales included in this study are part of the Bering-Chukchi-Beaufort population and their migration patterns take them between the Beaufort and Bering seas as the polar icecap grows and wanes throughout the year [25]. Beluga whales included in this study are from the Eastern Beaufort population, inhabiting the same general location and following a migration pattern similar to that of the bowheads [26]. Whales were caught as part of native Inupiat whale subsistence hunts. All tissues were collected from whales that were already deceased and none of the authors took part in any aspect of the hunting of the whales. All samples were taken on site either at Barrow Alaska (bowhead samples) or Point Lay (beluga samples) with full cooperation of local Inupiat hunters and captains and immediately stored in RNAlater® (Ambion) prior to storage at −80°C.

### RNA Isolation & cDNA Synthesis

Total RNA was extracted from approximately 100 mg of each tissue under RNAse-free conditions (RNAse OUT™, GBiosciences) using TRI-Reagent® (Ambion) manufacturer recommended protocols for high fat content samples and quantified via a Nanodrop® spectrophotometer ND-1000 (Nanodrop). RNAs were PCR analyzed for DNA contamination using designed primers LEPCDNAF and LEPCDNAR (Table 1) that span the leptin intron-exon border in an Eppendorf Personal minicycler (Eppendorf) under the following conditions: initial denaturation at 94°C for 2 minutes; 40 cycles of 94°C for 45 seconds, 54°C for 45 seconds, 70°C for 2 minutes for 1 final extension at 68°C for 10 minutes. Following DNA contamination testing, cDNA and no template controls (no RT) were synthesized using the High Capacity cDNA Reverse Transcription kit (Applied Biosystems) with manufacturer recommended protocols. All cetacean tissue cDNAs were normalized to 50 ng total RNA.

**Table 1:** Real-time primers for measuring expression of 18S, Uxt, Rs9, Rs15, leptin and OB-RGRP in cetacean tissues.

Gene	Primers	Sequence
18S	18SFReal	5′:AACCCGTTGAACCCCATT:3′
	18SRReal	5′:CCATCCAATCGGTAGTAGCG:3′
Uxt	Uxt323f	5′:TGTGGCCCTTGGATATGGTT:3′
	Uxt423r	5′:GGTTGTCGCTGAGCTCTGTG:3′
Rs9	Rs9192f	5′: CCTCGACCAAGAGCTGAAG:3′
	Rs9254r	3′:CCTCCAGACCTCACGTTTGTTC:3′
Rs15	Rs15405f	5′:GCAGCTTATGAGCAAGGTCGT:3′
	Rs15555r	5′:GCTCATCAGCAGATAGCGCTT:3′
DNA contamination check	LEPCDNAF	5′:CTCATCAAGACGATTGTCACCAGG:3′
	LEPCDNAR	5′:CAGCTGCCGCAGCATCGTCCTG:3′
leptin	Lep92F	5′:CCAAAACCCTCATCAAGACGAT:3′
	Lep325R	5′: GGAGAAGGTCCCGGAGGTT:3′
	Lep258F	5′:CACCAGTCTGCCTTCCAGAAAT:3′
OB-RGRP	GRP1F	5′:GAGACATGGCTGGCGTTAAAG:3′
	GRP750R	5′:AATAAGCCAGTTCCCGACAGGC:3′
	GRP620F	5′:CTGGTATGTGCCTTAGAGGATC:3′

### Relative Expression qPCR Assays

Endogenous internal control genes 18S, ubiquitously expressed transcript (Uxt), ribosomal protein 9 (Rs9) and ribosomal protein 15 (Rs15) were amplified from cetacean cDNA using designed primers (Sigma) based on previously published Genbank sequences (Table 1). PCR amplification of endogenous control products for UXT, Rs9 and Rs15 were completed in an Eppendorf Personal minicycler (Eppendorf) under the following conditions: initial denaturation at 94°C for 2 minutes; 30 cycles of 94°C for 30s, 50°C for 30s, 70°C for 2 minutes and final extension at 72°C for 5 minutes. Conditions for 18S amplification were as follows: initial denaturation at 94°C for 2 minutes; 30 cycles of 94°C for 30s, 60°C for 30s, 70°C for 2 minutes and final extension at 72°C for 5 minutes.

Leptin and OB-RGPR were amplified from cetacean cDNA using designed primers LEP92F and LEP325R and GRP1F and GRP750R, respectively (Table 1). PCR amplification of leptin and OB-RGRP products were done in an Eppendorf Personal minicycler (Eppendorf) under the following conditions: initial denaturation at 94°C for 2 minutes; 30 cycles of 94°C for 30s, 55°C for 30s, 70°C for 2 minutes and final extension at 72°C for 5 minutes.

All PCR results were evaluated with gel electrophoresis with subsequent ethidium bromide staining. Positive results for each gene were cloned into pCR®4-TOPO® vector using a TOPO TA Cloning® kit (Invitrogen™) following manufacturer recommended protocols and plated on X-Gal-treated LB/AMP plates. Colonies were PCR checked for correct insertion and plasmid preparations completed using QIAprep® Spin Miniprep Kit (Invitrogen™). Plasmid preparations were quantified via Nanodrop® spectrophotometer as previously described and sequenced using BigDye® Terminator v3.1 Cycle Sequencing kit and an ABI 3130xl genetic analyzer (Applied Biosystems, Foster City, CA) to confirm sequence identities prior to real-time PCR assays.

Relative qPCR assays were performed with iTaq™ SYBR® green Supermix with ROX (Biorad). Assays utilized designed leptin-specific qPCR primers Lep258F and Lep325R and OB-RGRP-specific real-time primers GRP620F and GRP750R and endogenous control gene primers for 18S, Rs9, Rs15 and Uxt described previously (Table 1). 20 µl reactions for all standards were run in triplicate with no template and primer controls on an ABI 7300 (Applied Biosystems) under the following conditions for leptin and OB-RGRP with 18S endogenous control: 60°C for 2 minutes, 95°C for 10 minutes, 95°C for 15 sec for 40 cycles, 60°C 1 minute. Assays of leptin and OB-RGRP with Rs9, Rs15 and Uxt endogenous controls were run under the following conditions: 55°C for 2 minutes, 95°C for 10 minutes, 95°C for 15 sec for 40 cycles, 50°C 1 minute. Results were analyzed following the 2^ΔΔCT^ method as described by Bustin [15].

### Construction of Bacterial Plasmid Standards for Validation of Absolute Quantification Real-time PCR Assays

Plasmid preparations for vectors containing confirmed inserts for cetacean leptin and OB-RGRP were first analyzed to examine plasmid structure (circular and linearized plasmid). Bacterial plasmids containing either leptin or OB-RGRP were either retained in circular form or linearized through restriction enzyme digestion using *Pst1* enzyme (Promega) and manufacturer recommended protocols. Completion of digestion was visualized through gel electrophoresis with ethidium bromide staining as previously described against non-digested plasmids prior to final quantification via Nanodrop® spectrophotometer. qPCR assays to determine the effect of linear versus circular plasmid structure on copy number estimation and amplification efficiencies were then conducted as described below.

The second validation test conducted assessed the importance of precipitating the plasmid standards prior to qPCR analysis. Digested plasmids (digestion described above) were then either used directly in qPCR analysis (for un-precipitated assays) or ethanol precipitated prior to quantification via Nanodrop® spectrophotometer.

The third validation test was performed using *Pst1* linearized plasmid standards for leptin and OB-RGRP to examine the effects of background material (either yeast RNA or cDNA) on amplification efficiencies and copy number estimations. Here, the linearized plasmid standards (described above) included either whole yeast RNA or cDNA as background in qPCR assays described below.

Copy number of all bacterial plasmids containing confirmed cetacean leptin and OB-RGRP inserts used in validation test were determined using the following formula: number = (ng * number/mole)/(bp * ng/g * g/mole of bp) (cite website: http://www.uri.edu/research/gsc/resources/cndna.html). For all qPCR assays described below for both genes, the bacterial plasmid standards spanned the full range of copy numbers found in cetacean tissues.

### Standard Curve Development and qPCR Analysis

The influence of bacterial plasmid structure, circular vs. restriction enzyme linearized plasmid, was examined via construction of a ten point standard curve for leptin ranging from 10∧5–100 copies per µl and for OB-RGRP a six point curve from 5.0*10∧3–100 copies per µl. In order to assess the importance of precipitation of restriction enzyme digested bacterial plasmid standards in copy number determination, a seven point standard curve for leptin ranging from 1.52*10∧5–5 copies per µl and a five point standard curve for OB-RGRP ranging from 10575-21 copies per µl were constructed. Finally, to ascertain the best type of background material, RNA or cDNA, for use in absolute quantification and evaluate the effect of this background material on copy number determination, a ten point curve was developed for both leptin and OB-RGRP ranging from 10∧5–100 copies per µl with 50 ng total of either starting yeast RNA or yeast cDNA.

As previously described, all qPCR assays used in this study employed the standards of known copy number described above and were performed with iTaq™ SYBR® green Supermix with ROX (Biorad), designed leptin-specific qPCR primers Lep258F and Lep325R and OB-RGRP-specific real-time primers GRP620F and GRP750R. As before, 20 µl reactions for all standards were run in triplicate with no template controls and primer controls on an ABI 7300 (Applied Biosystems) under the following conditions for all leptin plasmid validation tests: 60°C for 2 minutes, 95°C for 10 minutes, 95°C for 15 sec for 40 cycles, 60°C 1 minute. All OB-RGRP qPCR validation assays were run under the following conditions: 55°C for 2 minutes, 95°C for 10 minutes, 95°C for 15 sec for 40 cycles, 60°C 1 minute. Average Ct values and log (copy number) values were used to obtain a linear regression equation allowing for calculation of copy number of leptin and OB-RGRP in cetacean tissues. The efficiency was calculated using the following equation: Efficiency = ((10∧–1/slope)–1) [27].

### Construction of Bacterial Plasmid Standards for Absolute Quantification qPCR Assays of Endogenous Controls

Endogenous controls 18S, Rs9, Rs15 and Uxt were selected based on previously published mammalian real-time studies which utilized these genes for normalization [28], [29], [30], [31], [32], [33]. Plasmid preparations for vectors containing confirmed inserts for 18S, Rs9, Rs15 and Uxt were linearized via using Pst1 enzyme (Promega) and completion of digestion was visualized through gel electrophoresis as described previously. Completely digested products were ethanol precipitated prior to final quantification via Nanodrop® spectrophotometer and copy numbers of digested and precipitated bacterial plasmids standards for each gene were determined as previously described. For all four endogenous control gene standards, a six point standard curve was constructed. For 18S, the standard curve ranged from 6.0 * 10∧7–6.0*10∧5 copies per µl. For Rs9, the standard curve ranged from 5.95*10∧7–2.0*10∧5 copies per µl. For Rs15, the range covered by the standard curve included 5.0* 10∧7–1.5*10∧5 copies per µl and for Uxt this range included 6.45*10∧7–2.5*10∧5 copies per µl. qPCR assays were performed using iTaq™ SYBR® green Supermix with ROX (Biorad), gene-specific primers described above (Table 1) and 20 µl reactions for all control gene standards were run in triplicate with no template controls and primer controls on an ABI 7300 (Applied Biosystems) under previously described conditions for each gene. Linear regression equations and amplification efficiencies were calculated as described above.

### qPCR Analysis of Cetacean cDNAs

qPCR assays of bowhead and beluga tissue cDNAs was performed with iTaq™ SYBR® green Supermix with ROX (Biorad),leptin, OB-RGRP or endogenous control primers previously described (Table 1) under conditions described above. 20 µl reactions were run for each tissue in duplicate, with 50 ng total RNA used as template, alongside duplicate reactions of no template (no-RT) controls for each tissue and primer controls for each gene assayed. For all reactions, 50 ng total yeast cDNA was used as the background. Assays were run on an ABI 7300 (Applied Biosystems) under the previously described cycling conditions for all genes. Resulting Ave Ct values for each tissue were used in conjunction with the various gene-specific linear regression equations from standard curve analyses to calculate the copy number of each gene per 50 ng total RNA for each cetacean tissue assayed.

### Determination of Threshold Cycle, qPCR Amplification Efficiencies and Copy Number Determination

Threshold cycle values were calculated using Sequence Detection Software version 1.4 (Applied Biosystems) in conjunction with the ABI 7300 (Applied Biosystems). Standard curves for all qPCR assays for all genes and validation tests conducted were produced via linear regressions using threshold cycle (Ct) values and log_10_ starting copy number for each standard. Amplification efficiencies for all assays were calculated manually using the following equation: efficiency = 10^(−1/slope)^–1 [27]. Copy numbers were determined for each test and gene assayed based upon linear regression equations from standard curve assays for the respective test. Significance of copy number differences was based upon 95% confidence interval analyses and threshold cycle value significance was calculated using analysis of variance calculations (ANOVA).

## Results

### Effects of Four Endogenous Controls on Relative Expression of Cetacean Leptin and OB-RGRP

**Figure 1:**
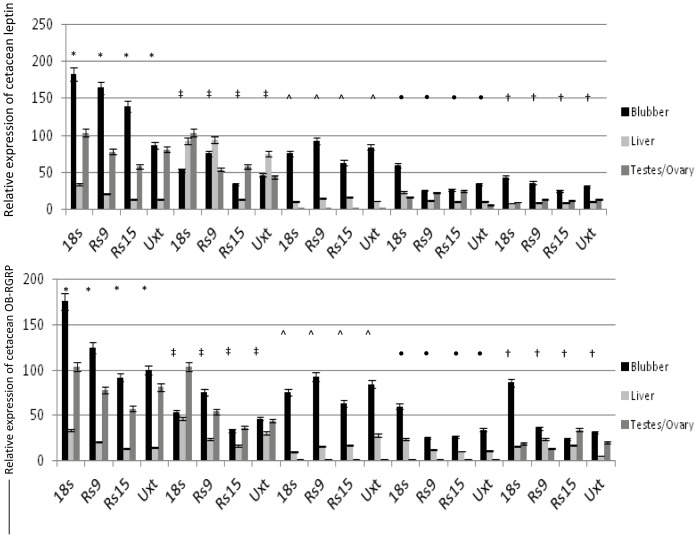
Relative expression values of cetacean leptin and OB-RGRP. Relative expression was calculated in three tissues from five different bowhead whales using four different endogenous controls: 18S, Rs9, Rs15 and Uxt. Error bars depict 95% confidence interval. *: 2010B16, ‡: 2011B3, ∧: 2009B7, •2010B15, †: 2009B9.

The relative expression of two model genes, leptin and OB-RGRP, were examined using four common endogenous control genes, 18S ribosomal RNA, ubiquitously expressed transcript (Uxt), ribosomal protein 9 (Rs9) and ribosomal protein 15 (Rs15). Three tissues from five individual bowhead whales were examined to assess if relative expression values of these model genes varied depending on the control gene utilized. Differences were in fact observed confirming normalization with different controls results in differences in expression (Figure 1).

### Absolute Quantification Validation Tests

#### Examination of Plasmid Structure on qPCR Amplification Efficiencies and Copy Number Determination

**Figure 2:**
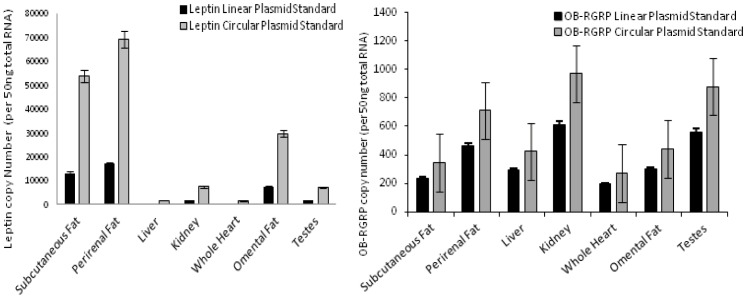
Copy number estimations from plasmid structure tests for OB-RGRP and leptin. Graphs represent copy numbers of leptin and OB-RGRP determined using circular and linearized plasmid standards. Note that copy numbers determined from linear and circular plasmid standards show significant difference. Error bars depict 95% confidence interval.

Standard curves for all methodology assays used in copy number estimation demonstrated high coefficients of determination (R^2^ values were between 0.9789–0.997 for all trials). Threshold cycle values for leptin circular plasmid standards ranged from 20.228 to 29.541 and linearized leptin plasmid standards for leptin ranged from 18.166 to 27.756 indicating no significant difference in threshold cycle values for the two plasmid structures (p = 0.128). Amplification efficiencies between circular and linearized leptin plasmids were also identical at 97% efficiency. The threshold cycle values for OB-RGRP circular plasmid standards ranged from 22.387 to 29.091 and threshold cycle values for linearized OB-RGRP plasmid standards ranged from 21.136 to 28.724. Again, no significant differences were seen in threshold cycle values when the two plasmid structures were compared (p = 0.557) and amplification efficiencies were nearly identical at 97.5% for circular OB-GRP plasmid and 97.7% for the linearized plasmid (Figure S1). Assays of bowhead whale tissues for both leptin and OB-RGRP using circular or linearized versions of the same plasmid standards consistently demonstrated higher threshold cycle (Ct) values for circular plasmid standards (Figure 2) regardless of the tissue assayed. For both genes assayed, significant copy number differences were seen when circular and linearized plasmid standards are used in standard curve assays. Estimated copy number values for bowhead whale tissues assayed using circular plasmid OB-RGRP standards ranged from 883 to 201 copies per 50 ng total starting RNA and copy number estimates for the same tissues and gene using linearized plasmid standard ranged from 609 to 194 copies per 50 ng total starting RNA. Estimates of copy number for leptin using circular plasmid standards ranged from 69246 to 1512 copies per 50 ng total starting RNA and copy number estimates using linearized plasmid standard ranged from only 16855 to 301 copies per 50 ng total starting RNA.

#### Examination of Plasmid Purity on qPCR Amplification Efficiencies and Copy Number Determination

**Figure 3:**
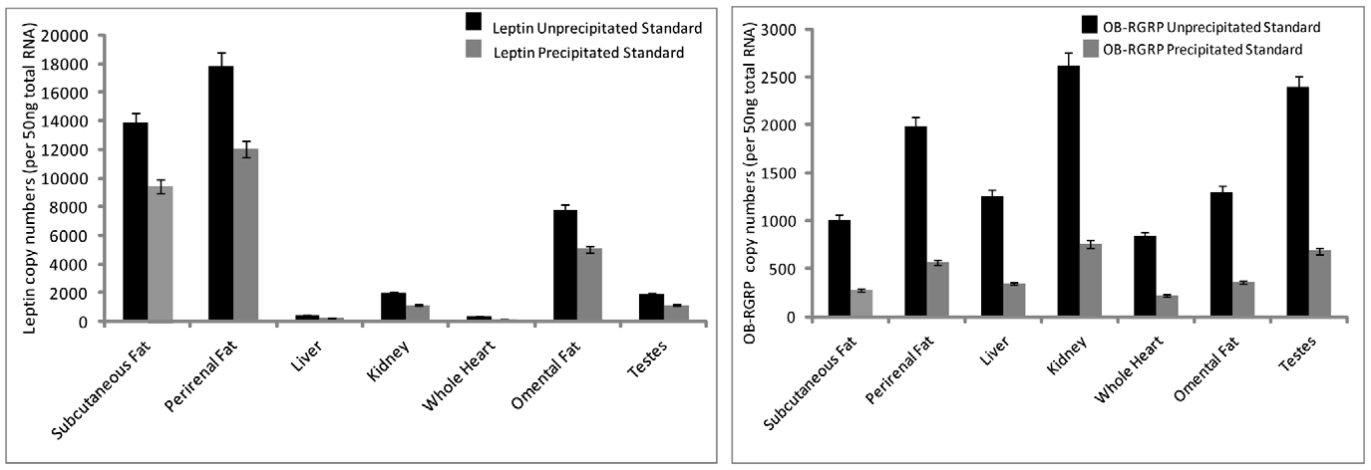
Copy number estimations from qPCR background material tests for OB-RGRP and leptin. Graphs represent copy numbers of leptin and OB-RGRP determined using yeast RNA and yeast cDNA as qPCR background material. Note that copy numbers determined from yeast RNA and yeast cDNA plasmid standards show no significant difference. Error bars depict 95% confidence interval.

Leptin plasmid threshold cycle values for validation tests of plasmid purity ranged from 17.878 to 33.030 for non-precipitated, linearized plasmid standards and from 16.936 to 31.255 for precipitated, linearized plasmid standards. Results indicate no significant differences in threshold cycle values between un-precipitated and precipitated leptin plasmids (p = 0.801). For OB-RGRP, threshold values for non-precipitated, linearized plasmid standards ranged from 22.295 to 34.067 while precipitated, linearized plasmid threshold values ranged from 19.876 to 31.435. As with leptin, the difference in threshold values between the two plasmid preparations yielded no significant differences (p = 0.438) (Figure S2). Assays of bowhead whale tissues for both leptin and OB-RGRP using precipitated or un- precipitated versions of the same plasmid standards consistently demonstrated higher threshold cycle (Ct) values for un- precipitated plasmid standards (Figure 3) regardless of the tissue assayed. Results of precipitation trials for both leptin and OB-RGRP demonstrated significant copy number differences based on plasmid standard preparation. Substantial overestimation of copy number was seen when un-precipitated standards are used in place of precipitated standards**.** Estimated copy number values for bowhead whale tissues assayed using un-precipitated plasmid OB-RGRP standards ranged from 2394–839 copies per 50 ng total starting RNA and copy number estimates for OB-RGRP using precipitated, linearized plasmids ranged from 759–224 copies per 50 ng total starting RNA. For leptin, copy number estimations using un-precipitated plasmid standards ranged from 17853 to 374 copies per 50 ng total starting RNA and estimates of copy number for tissues using precipitated plasmid standards ranged from 12023 to 201 copies per 50 ng total starting RNA.

#### Examination of Background on qPCR Amplification Efficiencies and Copy Number Determination

**Figure 4:**
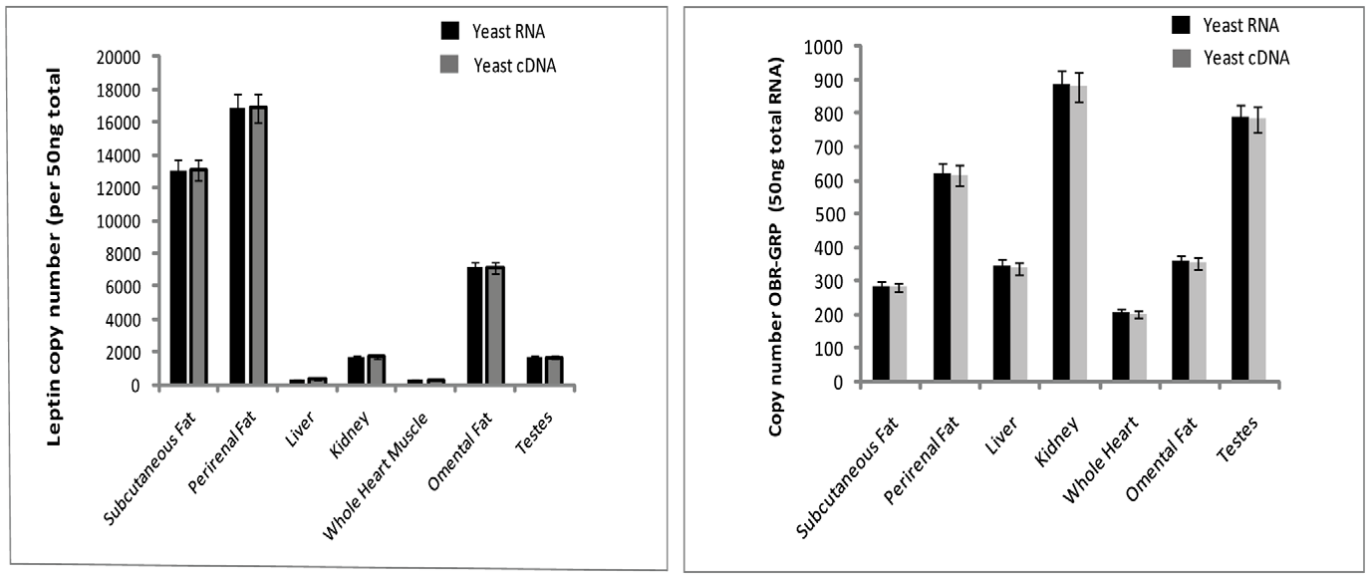
Copy number estimations from qPCR background material tests for OB-RGRP and leptin. Graphs represent copy numbers of leptin and OB-RGRP determined using yeast RNA and yeast cDNA as qPCR background material. Note that copy numbers determined from yeast RNA and yeast cDNA plasmid standards show no significant difference. Error bars depict 95% confidence interval.

Leptin threshold cycle values using RNA as background ranged from 18.169 to 27.787 and threshold cycles for trials using cDNA as background ranged from 18.166 to 27.756; demonstrating no significant difference in values based on background material selected (p = 0.998). For OB-RGRP, threshold cycle values ranged from 17.722 to 28.049 using RNA as background and 17.812 to 28.091 using cDNA as background with no significant difference seen (p = 0.987) (Figure S3). No significant difference in copy number estimations was seen for either leptin or OB-RGRP when either RNA or cDNA was used as a background (Figure 4). Furthermore, comparisons of standard curve assays demonstrate that standard curve points for both RNA and cDNA “background” often overlap for both genes. Copy number estimates for OB-RGRP for assays using RNA as background ranged from 885 to 206 copies per 50 ng total starting RNA and estimations for OB-RGRP copy number using cDNA as background ranged from 879 to 200 copies per 50 ng total starting RNA. For leptin, estimates of copy number for bowhead tissues using RNA as background ranged from 16858 to 299 copies per 50 ng total starting RNA and leptin copy number estimates using cDNA as background ranged from 16885 to 301 copies per 50 ng total starting RNA.

### Copy Number Determination of Leptin and OB-RGRP from Cetacean Tissues

**Figure 5:**
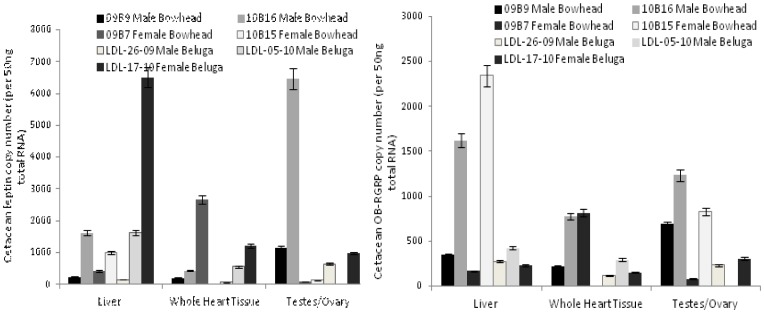
Copy numbers determine by absolute quantification real-time qPCR using precipitated, linearized plasmid standards for leptin and OB-RGRP. Graphs illustrate copy numbers differences in expression for both genes in liver, whole heart muscle and testes/ovary of two male bowheads (2009B9 and 2010B16), two female bowheads (2009B7 and 2010B15) as well as two male (2009LDL26 and 2010LDL05) and a single female (2010LDL17) beluga. Error bars depict 95% confidence interval.

The use of absolute quantification real-time qPCR assays allow for comparisons to be drawn between individuals and among individuals of different species. Assays were conducted to examine copy number variations in two model genes, leptin and OB-RGRP, between individuals of two species, the bowhead (*Balaena mysticetus*) and beluga (*Delphinapterus leucas*) whale (Figure 5). Standard curves used for calculations of copy number of both genes employed the rigorously tested methodology described above with linearized, precipitated plasmids and yeast cDNA background material. Results demonstrate differences between tissues, between individuals and between species for both genes. Overall, higher expression values are seen for leptin compared to OB-RGRP when the same total amount of RNA is used in the qPCR assays.

### Copy Number Determination of Endogenous Controls 18S, Rs9, Rs15 and Uxt from Cetacean Tissues

**Figure 6:**
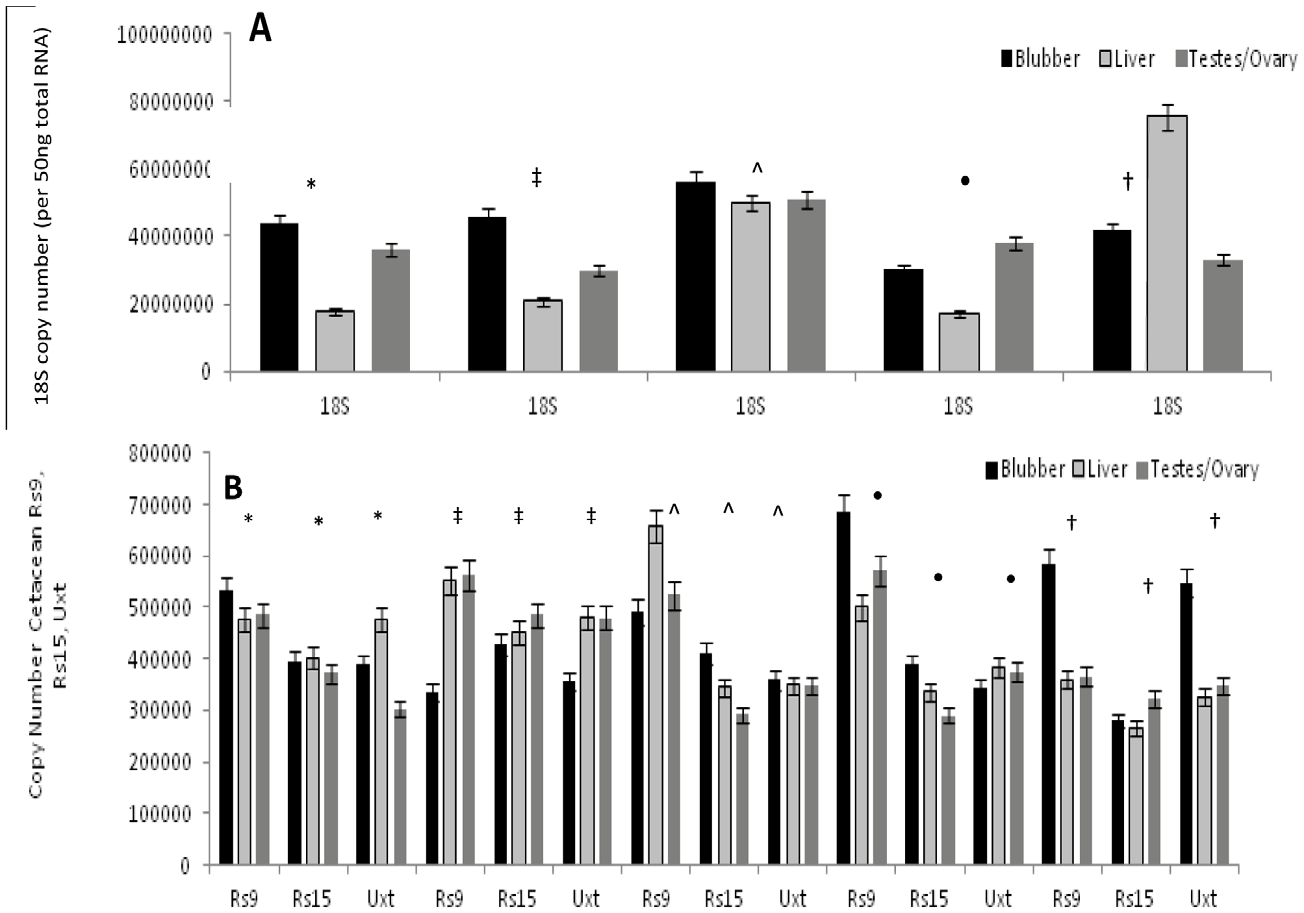
Copy numbers determine by absolute quantification real-time qPCR using precipitated, linearized plasmid standards for (A) 18S and (B) Rs9, Rs15 and Uxt. Graphs illustrate copy numbers differences in expression in blubber, liver and testes/ovary of two male bowheads (2009B9 and 2010B16), and three female bowheads (2010B15, 2011B3 and 2009B7). Error bars depict 95% confidence interval. *: 2010B16, ‡: 2011B3, ∧: 2009B7, •: 2010B15, †: 2009B9.

We applied absolute quantification methods described above to establish standard curves specific to each of the four endogenous control genes used in the relative expression assay (18S, Rs9, Rs15 and Uxt). Threshold values ranged from 11.672 to 20.152 for 18S, 11.455 to 20.153 for Rs9, 11.288 to 19.194 for Rs15 and 11.431 to 19.078 for Uxt. No significant difference was seen in the threshold cycle values for the endogenous control genes (p = 0.990) (Figure S4). Significant differences in copy number were observed among tissues surveyed and between those tissues in individual whales. Furthermore, the number of copies expressed per 50 ng total starting RNA varied greatly between the genes, resulting in an approximately 10 fold difference seen in the number of copies expressed for 18S compared to Rs9, Rs15 and Uxt expression levels (Figure 6).

## Discussion

Examination of physiological systems in marine mammals is complicated by both the life histories of the study animals and access to viable samples. In recent years, relative qPCR assays have been used in marine mammal studies to probe gene expression and provide information regarding ecotype, immunological assessments and physiological response to both metal sensitivity and various pollutants [34], [35], [36], [37]. Our interests here are in the pattern and expression of two genes, leptin and OB-RGRP in two species of arctic adapted cetacean: the bowhead (*Balaena mysticetus*) and beluga (*Delphinapterus leucas*) whale. Leptin was of particular interest in these species since it is primarily synthesized by adipose cells and these species build and maintain large subcutaneous fat reserves (blubber) throughout their lifetime [1]. Do these large adipose stores actually correlate to high leptin mRNA expression in these species? Relative qPCR assays would address this question among individuals and tissues on a single plate, but only absolute quantification methods would allow us to address this question among individuals *and* the two different species.

### Relative versus Absolute Estimations of Endogenous Controls

Currently, the most commonly used method in real-time qPCR assays is relative quantification, but these assays are limited by not permitting comparisons to be drawn between samples run at different times or between species. Moreover, using this method target gene amplification is normalized to exogenous or endogenous controls *assumed* to demonstrate no difference is expression regardless of age, sex or treatment [15]. Recent work has raised questions about these assumptions by demonstrating that many commonly utilized controls do demonstrate significant changes in expression level and pattern in among tissues in a variety of taxa [15], [18], [19], [20], [21]. Here, we sought to examine what effect, if any, the choice of endogenous control gene had on the relative expression of two model target genes, leptin and leptin-receptor gene-related product (OB-RGRP), in a species where very little is known regarding control gene levels and patterns of expression: the bowhead whale (*Balaena mysticetus*). We normalized amplification of these two model target genes with that of four different endogenous control genes employed previously in relative qPCR studies of other mammalian taxa: 18S ribosomal RNA, ubiquitously expressed transcript (Uxt), ribosomal protein 9 (Rs9) and ribosomal protein 15 (Rs15). Multiple tissues from five individual whales were assayed and results demonstrated significant differences in expression for both genes between both different tissues and different individuals when normalized using the different endogenous controls (Figure 1). This supports earlier findings that variation in endogenous control gene expression does influence relative expression qPCR results.

We applied the absolute quantification methodology described here to analyze the levels and patterns of the same four endogenous control genes discussed previously (18S, Rs9, Rs15 and Uxt) to determine if this method could also detect significant differences in copy number in the four genes. Whereas no significant differences were observed in the threshold cycle values for the endogenous controls (p = 0.990), significant differences in copy number were observed among tissues and individuals in the three tissues surveyed. There was also an approximately 10 fold difference seen in the number of copies expressed for 18S compared to Rs9, Rs15 and Uxt expression levels (Figure 6). Of the four genes assayed, it appears that Rs15 more consistently demonstrated the smallest range of variation between the three tissue types and amongst individuals are therefore more likely to demonstrate true variations between those genes whereas data obtained using variable control genes will result in larger estimated differences (Figure 6).

### Validations of Absolute qPCR Methodology

“Absolute” qPCR is a second method of analyzing target gene expression. This method forgoes relative expression analysis in favor of measuring actual copy number values via the construction of a standard curve from a variety of different sources [22]. This method requires more initial preparation, but provides the important added benefit of allowing for direct inter- and intra-specific comparisons of expression levels of specific targets. This permits comparisons of copy numbers and expression profiles from species of interest with those of more commonly used model organisms –comparisons not possible using relative expression analysis. Here, we evaluated the methods used in absolute quantitative qPCR through extensive validation tests. Using two model genes, leptin and OB-RGRP, we analyzed differences in copy number estimation and amplification efficiencies based on several variables to ascertain that the method reported is valid and provides highly sensitive and reproducible results.

A recent study examined gene copy number variation of the *pcna* gene in various algal species and determined that there was a significant difference in copy number depending on the structure of the plasmid used to construct the standard curve, whether it was circular or enzymatically linearized [22]. We examined this effect of plasmid structure on leptin and OB-RGRP expression in various cetacean tissues through the use of both circular and linearized plasmids in absolute quantification qPCR assays. The data show no significant difference in amplification efficiencies between the two plasmid structures assayed (circular versus linearized) for both leptin and OB-RGRP; with approximately parallel slopes and p values of 0.128 and 0.557, respectively (see Figure S1). Despite these similar amplification efficiencies, our data do confirm that there were significant differences in the estimated copy numbers in many tissues depending on the structure of plasmid used (circular or linearized) for both genes in the cetacean tissues (Figure 2).

Validation tests were also conducted to examine the possible effects of plasmid standard purity (precipitated versus un-precipitated linearized standards) on copy number determination and amplification efficiency of linearized bacterial plasmids of cetacean leptin and OB-RGRP. As with the plasmid structure tests, regression curves for both circular and linearized plasmid showed parallel slopes and demonstrated no significant difference in amplification efficiencies with p values of 0.801 (leptin) and 0.438 (OB-RGRP). Results demonstrated a similar pattern of expression in both genes when both the un-precipitated and precipitated standards were used, but significant overestimations of copy number were detected in several tissues for both genes in the cetacean tissues (Figure 3) when un-precipitated standards were used in comparison to precipitated standards.

The set-up of any qPCR standard run, whether for use in standard curve development or for tissue cDNA analysis, should include background genetic material that functions to mimic natural conditions normally found *in vivo*. To determine the best method for achieving these conditions, validation tests were conducted using both total RNA and total cDNA as “background” material to determine what effect, if any, this change had on qPCR amplification efficiencies and copy number determination of leptin and OB-RGRP from cetacean tissues. It is worth noting that the source of this “background” material must be carefully selected to insure the absence of any target gene from background source to avoid any co-occurring amplification. In our assays, we employed either total RNA or total cDNA (both extracted from yeast tRNAs) which lack coding sequences for either leptin or mammalian OB-RGRP. Regression curves for both genes yielded nearly identical slopes and p values of 0.998 (leptin) and 0.987 (OB-RGRP) indicative that the choice of RNA or cDNA as background made no significant changes to amplification efficiencies in either gene (Figure 5). Additionally, no significant differences in copy number determination were seen in examinations of leptin or OB-RGRP in any tissue surveyed (Figure 4). This data demonstrates the high degree of reproducibility possible with absolute quantification analyses and suggests that the use of either total RNA or cDNA as background material is acceptable for set-up of absolute quantification qPCR standard assays and does not significantly affect results obtained.

### Conclusions

Our interests lie in determining not only the pattern, but the level of an adipose-derived protein (leptin) and a related protein (OB-RGRP) in various tissues of two species of arctic adapted cetacean. Data acquired using only relative qPCR methods provide us only with the former. Data reveals that both species of cetacean produce leptin and that, like other mammals, they produce more leptin in their adipose tissues compared to other tissues (unpublished data). The unique size and extent of cetacean adipose depots, however, makes the question of *total* expression one of significance to cetacean biology. Only absolute qPCR methodology allows us to measure levels of expression via actual copy numbers of mRNAs and compare those values among individuals and among species. Figure 5 illustrates significant differences in copy number among individuals in the three tissues surveyed for both genes in both species of cetacean. Future studies incorporating more individuals and tissues from both species will allow us to compare levels and patterns of leptin and OB-RGRP expression and examine seasonal, ontogenetic and sex-specific differences in these cetacean species. Doing so using absolute qPCR methods will also allow us to compare these cetacean values (from an animal that depends highly on maintained adipose reserves) to those attained from closely related ungulates and other mammals.

Our data suggests that individual and sex-specific differences may occur in the expression of the two model genes and that expression levels vary (per 50 ng starting RNA) for the two genes. Furthermore, data regarding the pattern and level of expression for the four endogenous controls demonstrates the range of variation and fold differences seen in between these genes. The absolute quantification methodology described and validated here provides a method for determination of mRNA copy number for genes of interest in any physiological pathway. This technique should prove especially useful in cetacean studies: allowing for examination of a plethora of physiologically relevant genes using only small amounts of starting materials from access to recently dead specimens or non-invasive biopsies or sera samples.

With respect to alternative qPCR methodologies, our results suggest that with the correct precautions the use of absolute quantification can provide both more accurate expression data in addition to those data being more useful in comparative studies. We have provided validation tests to demonstrate this “absolute” qPCR method reported is valid and provides highly sensitive and reproducible results. We examined the effects of several different variables on the amplification efficiencies and copy number determination of two model genes, leptin and OB-RGRP in various tissues from a bowhead whale in order to determine the best method for conducting absolute quantification qPCR analyses. Based on our results, the best means of standard curve preparation for use in absolute qPCR assays employs the use of a precipitated, enzymatically linearized plasmid containing sequence of a target gene of interest. Additionally, standard curve development and qPCR assays should ideally include the use of a “background” material to mimic natural conditions normally found *in vivo* and our data demonstrates that either RNA or cDNA templates are suitable for this purpose.

## Supporting Information

Figure S1 **Standard curves for linearization tests of leptin and OB-RGRP.** Linear regression lines illustrate differences in threshold cycles (Ct) between linear and circular plasmid standards for both leptin and OB-RGRP. Amplification efficiencies calculated using Efficiency =  ((10∧–1/slope)–1) [27].
(TIF)

Figure S2 **Standard curves for plasmid precipitation tests of leptin and OB-RGRP.** Linear regression lines illustrate differences in threshold cycles (Ct) between linear and circular plasmid standards for both leptin and OB-RGRP. Amplification efficiencies calculated using Efficiency =  ((10∧–1/slope)–1) [27].
(TIF)

Figure S3 **Standard curves for qPCR background material tests of leptin and OB-RGRP.** Linear regression lines illustrate no significant differences in threshold cycles (Ct) between yeast RNA and yeast cDNA background for both leptin and OB-RGRP. Amplification efficiencies calculated using Efficiency =  ((10∧–1/slope)–1) [27].
(TIF)

Figure S4 **Standard curves for qPCR analysis of 18S (A), Uxt (B), Rs9 (C) and Rs15 (D).** All linear regressions showed R^2^ values ranging from 0.9071-0.9915 and amplification efficiencies were calculated using Efficiency =  ((10∧–1/slope)–1) [27].
(TIF)
