# Supplementary figures and images for: Leptin in Whales: Validation and Measurement of mRNA Expression by Absolute Quantitative Real-Time PCR
Source: PLoS One. 2013 Jan 16;8(1):e54277. doi: 10.1371/journal.pone.0054277 (PMC3546977; doi:10.1371/journal.pone.0054277)

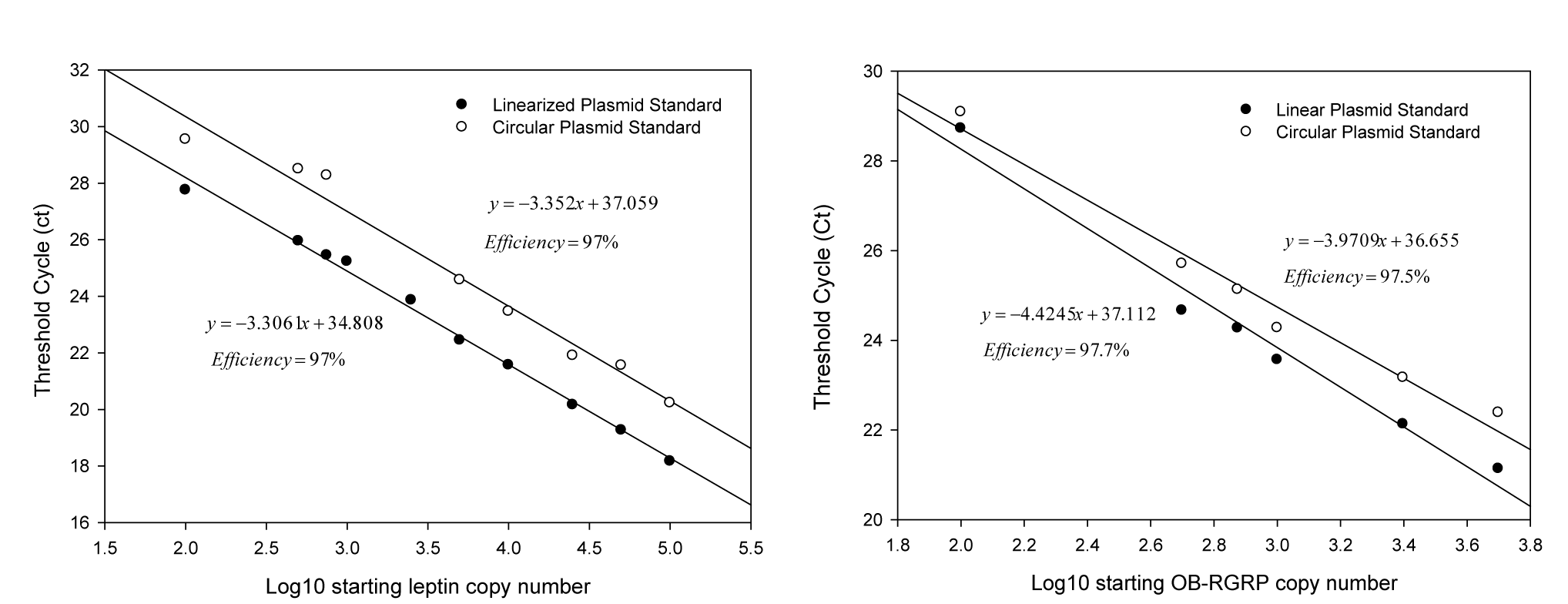

Supplement: Figure S1 — Standard curves for linearization tests of leptin and OB-RGRP. Linear regression lines illustrate differences in threshold cycles (Ct) between linear and circular plasmid standards for both leptin and OB-RGRP. Amplification efficiencies calculated using Efficiency = ((10∧–1/slope)–1) [27]. (TIF) [file pone.0054277.s001.tif]

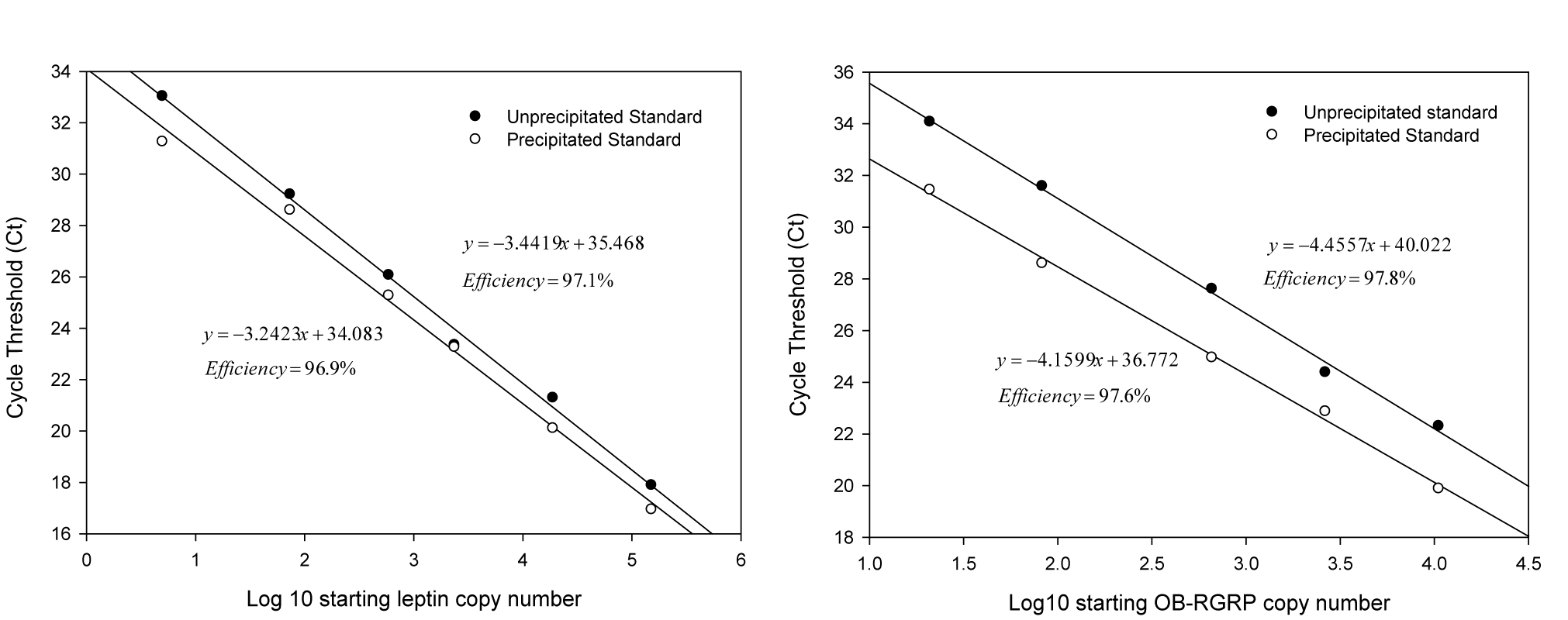

Supplement: Figure S2 — Standard curves for plasmid precipitation tests of leptin and OB-RGRP. Linear regression lines illustrate differences in threshold cycles (Ct) between linear and circular plasmid standards for both leptin and OB-RGRP. Amplification efficiencies calculated using Efficiency = ((10∧–1/slope)–1) [27]. (TIF) [file pone.0054277.s002.tif]

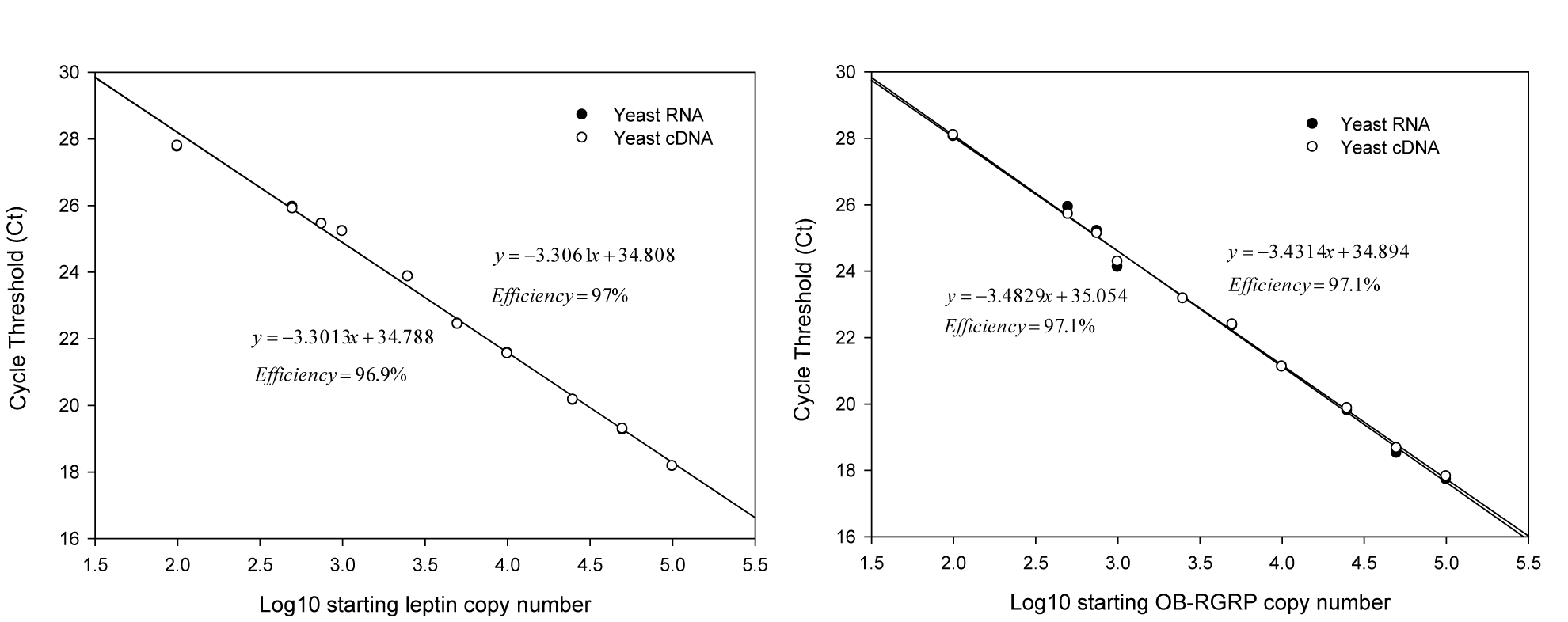

Supplement: Figure S3 — Standard curves for qPCR background material tests of leptin and OB-RGRP. Linear regression lines illustrate no significant differences in threshold cycles (Ct) between yeast RNA and yeast cDNA background for both leptin and OB-RGRP. Amplification efficiencies calculated using Efficiency = ((10∧–1/slope)–1) [27]. (TIF) [file pone.0054277.s003.tif]

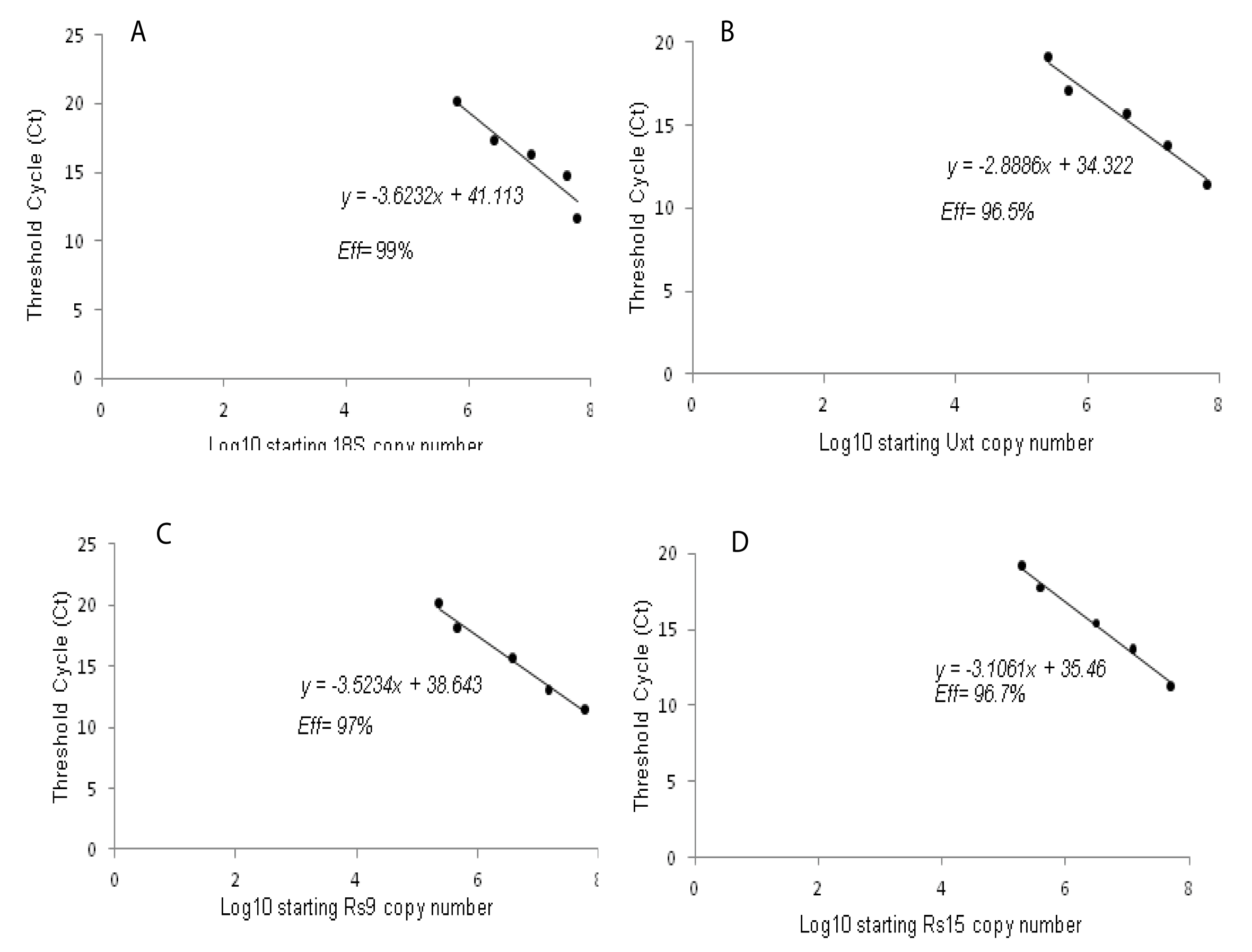

Supplement: Figure S4 — Standard curves for qPCR analysis of 18S (A), Uxt (B), Rs9 (C) and Rs15 (D). All linear regressions showed R2 values ranging from 0.9071-0.9915 and amplification efficiencies were calculated using Efficiency = ((10∧–1/slope)–1) [27]. (TIF) [file pone.0054277.s004.tif]
